# Molecular Basis of Cardiac and Vascular Injuries Associated With COVID-19

**DOI:** 10.3389/fcvm.2020.582399

**Published:** 2020-11-03

**Authors:** Mahmood Yaseen Hachim, Saba Al Heialy, Abiola Senok, Qutayba Hamid, Alawi Alsheikh-Ali

**Affiliations:** ^1^College of Medicine, Mohammed Bin Rashid University of Medicine and Health Sciences, Dubai, United Arab Emirates; ^2^Meakins-Christie Laboratories, Research Institute of the McGill University Health Center, Montreal, QC, Canada; ^3^College of Medicine, Sharjah Institute for Medical Research, University of Sharjah, Sharjah, United Arab Emirates

**Keywords:** COVID-19, SARS-CoV-2, cardiac and vascular injuries, transcriptomic, DEG (differentially expressed gene) analysis

## Abstract

**Background:** Coronavirus disease 2019 (COVID-19) is a viral respiratory illness caused by the novel coronavirus SARS-CoV-2. The presence of the pre-existing cardiac disease is associated with an increased likelihood of severe clinical course and mortality in patients with COVID-19. Besides, current evidence indicates that a significant number of patients with COVID-19 also exhibit cardiovascular involvement even in the absence of known cardiac risk factors. Therefore, there is a need to understand the underlying mechanisms and genetic predispositions that explain cardiovascular involvement in COVID-19.

**Objectives:** *In silico* analysis of publicly available datasets to decipher the molecular basis, potential pathways, and the role of the endothelium in the pathogenesis of cardiac and vascular injuries in COVID-19.

**Materials and Methods:** Consistent significant differentially expressed genes (DEGs) shared by endothelium and peripheral immune cells were identified in five microarray transcriptomic profiling datasets in patients with venous thromboembolism “VTE,” acute coronary syndrome, heart failure and/or cardiogenic shock (main cardiovascular injuries related to COVID-19) compared to healthy controls. The identified genes were further examined in the publicly available transcriptomic dataset for cell/tissue specificity in lung tissue, in different ethnicities and in SARS-CoV-2 infected vs. mock-infected lung tissues and cardiomyocytes.

**Results:** We identified 36 DEGs in blood and endothelium known to play key roles in endothelium and vascular biology, regulation of cellular response to stress as well as endothelial cell migration. Some of these genes were upregulated significantly in SARS-CoV-2 infected lung tissues. On the other hand, some genes with cardioprotective functions were downregulated in SARS-CoV-2 infected cardiomyocytes.

**Conclusion:** In conclusion, our findings from the analysis of publicly available transcriptomic datasets identified shared core genes pertinent to cardiac and vascular-related injuries and their probable role in genetic susceptibility to cardiovascular injury in patients with COVID-19.

**Graphical Abstract:**
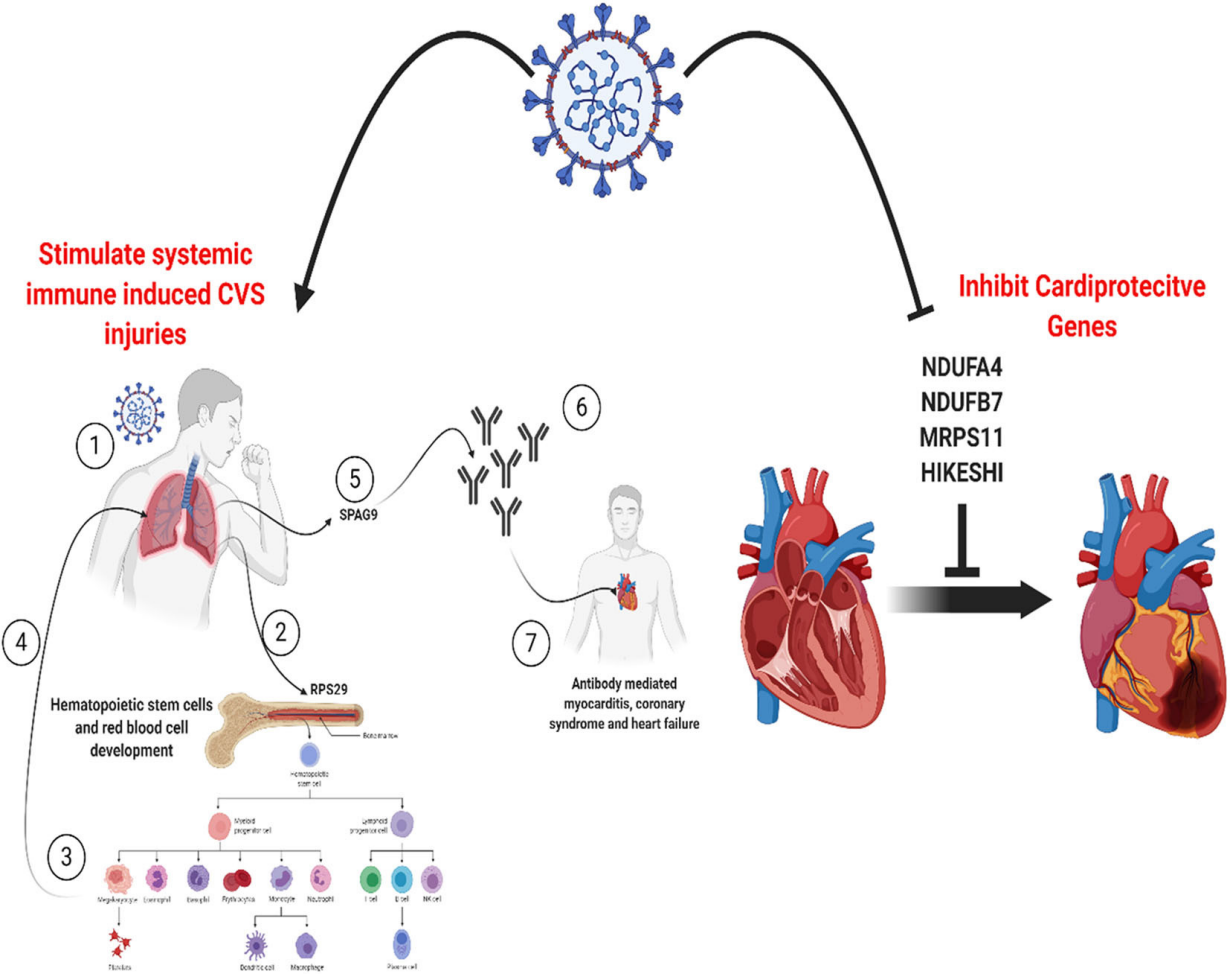
SARS-CoV-2 can induce cardiovascular injures in COVID-19 patients by manipulating a core set of genes specific to endothelium in the lungs, heart, and vessels. This can activate pathways for systemic immune-mediated cardiovascular injuries or increase vulnerability to cardiac injury via inhibition of cardioprotective proteins. Created with BioRender.com.

## Introduction

Coronavirus disease 2019 (COVID-19) is a viral respiratory illness caused by the novel coronavirus SARS-CoV-2. To date (1st September 2020), the number of laboratory-confirmed cases of COVID-19 has exceeded 25 million globally, with over 800,000 fatalities (1). The clinical spectrum of COVID-19 ranges from asymptomatic infection to mild to moderate disease in the majority of patients (2, 3). However, some patients exhibit a more severe clinical course characterized by multisystemic and life-threatening manifestations with pneumonia and acute respiratory distress as prominent features (2–4). Patients with pre-existing cardiac disease, hypertension, diabetes, and obesity are more likely to have a severe clinical course with a higher risk of mortality (5–7). In a meta-analysis of 8 studies, including 46,248 patients, cardiovascular disease was the third most common comorbidity in patients with COVID-19 (4). Moreover, there is increasing evidence that a significant number of patients with COVID-19 have cardiovascular involvement, which further increases the likelihood of mortality (5, 6, 8, 9). Notably, even in the absence of known cardiac risk factors, patients with COVID-19 may have an increased risk of cardiovascular injury with a report from China documenting high levels of troponin or cardiac arrest in up to 12% of patients without prior history of cardiovascular disease (6).

The acute cardiovascular syndrome associated with COVID-19 includes a variety of clinical presentations of acute cardiac injury, cardiomyopathy, and hemodynamic instability. Myocardial injury, arrhythmias, cardiac arrests, heart failure, and coagulation abnormality were reported in 7–33% of patients with COVID-19 in China (3, 9). The angiotensin-converting enzyme 2 (ACE-2) receptors used for cellular entry by SARS-CoV-2 are expressed in the lung as well as in various organs, including the heart and endothelial cells (10–12). Direct SARS-CoV-2 infection of the endothelial cells, along with diffuse endothelial inflammation, has been reported (11). The cytokine storm and profound inflammation seen in patients with severe COVID-19 are associated with macrophage and endothelial activation and surges in the levels of interleukin (IL)-1, IL-6, IL-8, and Tumor Necrosis Factor-alpha (TNF-α). Emerging data also indicate a hypercoagulable state in a cluster of patients with COVID-19 with a high incidence of venous thromboembolism (VTE) despite the use of prophylactic anticoagulants (13). Studies have shown that IL-6, one of the significant cytokines described in the cytokine storm, is associated with vascular leakage, activation of the coagulation cascade, and cardiomyopathy (14, 15).

One of the proposed mechanisms of cardiovascular injury in COVID-19 is direct injury to myocardial cells due to viral invasion of the vascular endothelium and myocardium (16). The second postulate is the impact of tissue hypoxia, destabilization of coronary plaque, and micro-thrombogenesis caused by the systematic inflammation associated with cytokine storm (16). In addition, the potential role of genetic susceptibility to COVID-19 related cardiac events has recently been highlighted as a possible contributor to the high mortality among African American patients with COVID-19 (17). As cardiovascular involvement in COVID-19 is now recognized as a predictor of mortality, there is a need to understand the underlying mechanisms and genetic predisposition.

Endothelial cells, like other structural cells, when physiologically activated or during injury like the case of cardiovascular diseases with or without COVID-19, can release increased levels of circulating phospholipid-rich microvesicles that can affect recipient cells locally or via the systemic circulation (18). Such vesicles, called exosomes, may enclose a range of parent cell molecules, including nucleic acids (DNA, mRNA, microRNA, and lncRNA), proteins, and lipids (19). Necrotic or apoptotic processes induced during vascular endothelium damage can lead to the dissemination of such exosomes such that mRNA detected in the circulation can be representative of cells that do not circulate (20). Sampling and molecular analysis of such circulating cells, extracellular vesicles, nucleic acids, which is referred to as liquid biopsy, is emerging as a promising approach for research in cardiovascular injuries (19). Recently endothelial, granulocyte, and platelet-derived exosomes were used to discriminate and map coronary atherosclerotic plaque and calcification in asymptomatic patients (21). In line with this paradigm, we carried out *in silico* analysis of publicly available datasets derived from different cell sources to decipher the molecular basis, potential pathways, and the role of the endothelium in the pathogenesis of cardiac and vascular injuries in COVID-19.

## Materials and Methods

### Identification of Differentially Expressed Genes in Blood Cells Following Cardiovascular Injuries

#### Datasets

Publicly available transcriptomic datasets were retrieved from Gene Expression Omnibus (GEO) (https://www.ncbi.nlm.nih.gov/geo/). Microarray gene expression datasets with the word “venous thromboembolism, acute coronary syndrome, arrhythmia, viral myocarditis, heart failure, and/or cardiogenic shock” were selected. Then we selected datasets with human patients' samples that were compared with age-matched healthy controls and where the samples studied were either whole blood, peripheral blood cells, or endothelium. No datasets of viral myocarditis or cardiogenic shock fulfilled these inclusion criteria. The five datasets (215 patients and 109 healthy control) that fulfilled the inclusion criteria are shown in Table 1.

**Table 1:** List and details of publicly available transcriptomic datasets used in the identification of differentially expressed genes (DEGs) in cardiovascular injuries.

**Cardiovascular injuries**	**Title**	**GSE**	**Sample type**	**Patients**	**Controls**
Venous thromboembolism	Whole blood gene expression profiles distinguish patients with single vs. recurrent venous thromboembolism	GSE19151	Whole blood	70	63
	Whole blood gene expression profiles distinguish clinical phenotypes of venous thromboembolism [Set1]	GSE48000	Whole blood	109	25
	Gene expression profile of endothelial colony-forming cells (ECFCs) isolated from patients with unprovoked venous thromboembolism (uVTE)	GSE118259	Endothelial cells	8	5
Acute coronary syndrome	Differential gene expression in thrombus-derived white blood cells of patients with acute coronary syndrome	GSE19339	White blood cells	4	4
Heart failure and/or cardiogenic shock	Expression data from heart failure vs. control peripheral blood mononuclear cells	GSE9128	Peripheral blood mononuclear cells	24	12
Total		5 datasets		215	109

### DEGs

We used GEOquery and limma R packages through the GEO2R tool for each dataset (22). We selected the differentially expressed probes, as previously described (23). Briefly, we sorted the genes related to the filtered probes according to the False Discovery Rate (FDR) and selected the top 2,000 differentially expressed probes with FDR <0.05 from each dataset. The annotated genes in each dataset were intersected with DEGs from all other datasets. Enriched Ontology Clustering for the identified genes was performed using the Metascape (http://metascape.org/gp/index.html#/main/step1).

### Identification of DEGs in Different Ethnicities

In light of the premise for a potential role for genetic susceptibility to cardiovascular injuries associated with COVID-19, we further explored for the expression of the identified DEGs in the publicly available dataset (GSE17078) of blood outgrowth endothelial cells from 27 healthy subjects of diverse ages and grouped into Caucasian and African Americans.

### Virus Perturbations From GEO

In order to explore if the identified genes showed differential expression during viral infections and to identify the viruses that affect their expression, we used the “Gene-virus associations by differential expression of gene following viral infection” database. “https://amp.pharm.mssm.edu/Harmonizome/dataset/GEO+Signatures+of+Differentially+Expressed+Genes+for+Viral+Infections.”

### Lung Gene Expression

To identify which lung cells specifically express the genes of interest at a significantly higher level compared to other cells, we explored LungGENS (Lung Gene Expression iN Single-cell), a web-based resource for querying lung single-cell gene expression databases (24).

### Identification of DEGs in SARS-CoV-2 Infected Cells and Lungs

The expression of the shortlisted genes was explored in the dataset (GSE147507), where RNA-sequencing of transformed alveolar lung cells (A549) were mock-treated (*n* = 6) or infected with SARS-CoV-2 (USA-WA1/2020) (*n* = 6) (25). The same dataset contains uninfected human lung biopsies, one male (age: 72 years), and one female (age: 60 years), which were used as biological replicates and were compared to lung samples derived from a single deceased male patient with COVID-19 (age: 74 years). The retrieved data were used to identify DEGs between infected and uninfected lung samples using BioJupies online tool (https://amp.pharm.mssm.edu/biojupies/). The normalized gene expression was used further to estimate infiltrating immune cells in the lungs.

### Estimation of Infiltrating Immune Cells in the Lungs

The normalized gene expression was uploaded to CIBERSORT (https://cibersort.stanford.edu/) to quantify immune cell fractions from bulk lung tissue gene expression profile (26).

### Map of Protein Expression Across Human Tissues

Tissue specificity of the identified genes was investigated using The Human Protein Atlas (https://www.proteinatlas.org/) (27). A blood cell-type expression (RNA) option was used to examine the cell specificity of the identified genes. Normalized expressions (NX) for 18 blood cell types and total peripheral blood mononuclear cells (PBMC) were explored.

### Identification of Differentially Expressed Genes in SARS-CoV-2 Infected Human-Induced Pluripotent Stem Cell-Derived Cardiomyocytes

The expression of the shortlisted genes was explored in the transcriptomic dataset “GSE150392” which is derived from human-induced pluripotent stem cell-derived cardiomyocytes infected *in vit*ro with SARS-CoV-2. The genes which showed significant differential expression between SARS-CoV-2 and mock-infected cells were identified.

## Results

### Whole Blood and Endothelium Shared DEGs in Patients With Venous Thromboembolism

**Figure 1:**
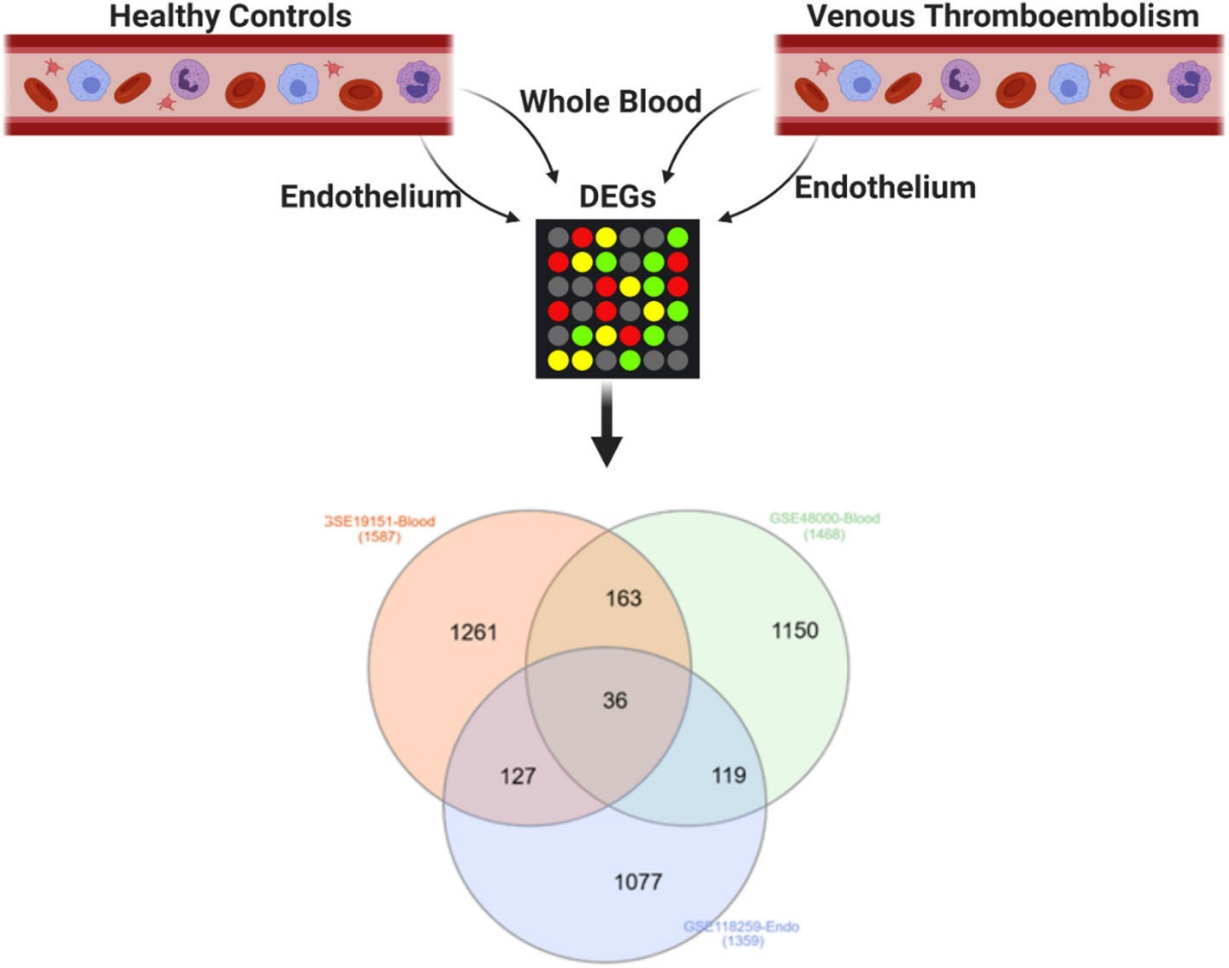
Shared DEGs in the blood and endothelium of patients with venous thromboembolism compared to healthy controls. DEGs in the whole blood of patients with venous thromboembolism (GSE19151 and GSE48000) were intersected with DEGs in endothelial cells of patients with venous thromboembolism (GSE118259). Created with BioRender.com.

DEGs in the whole blood of patients with VTE relative to healthy controls (GSE19151 and GSE48000) were intersected with DEGs in endothelial cells of patients with VTE relative to healthy controls (GSE118259), and 36 genes were identified as DEGs common to the three datasets, suggestive of their role in VTE (Figure 1, Table 2).

**Table 2:** List of the 36 shared DEGs in the blood and endothelium of patients with venous thromboembolism compared to healthy controls.

**Gene symbol**	**Description**	**Biological process (GO)**
*AFTPH*	Aftiphilin	GO:0015031 protein transport;GO:0015833 peptide transport;GO:0046907 intracellular transport
*AHNAK*	AHNAK nucleoprotein	GO:1901385 regulation of voltage-gated calcium channel activity;GO:1901019 regulation of calcium ion transmembrane transporter activity;GO:1903169 regulation of calcium ion transmembrane transport
*AQR*	Aquarius intron-binding spliceosomal factor	GO:0006283 transcription-coupled nucleotide-excision repair;GO:0006289 nucleotide-excision repair;GO:0000398 mRNA splicing, via spliceosome
*CHD9*	Chromodomain helicase DNA binding protein 9	GO:0032508 DNA duplex unwinding;GO:0032392 DNA geometric change;GO:0071103 DNA conformation change
*CNPY2*	Canopy FGF signaling regulator 2	GO:0045716 positive regulation of low-density lipoprotein particle receptor biosynthetic process;GO:0045714 regulation of low-density lipoprotein particle receptor biosynthetic process;GO:0010870 positive regulation of receptor biosynthetic process
*DEDD*	Death effector domain containing	GO:0008625 extrinsic apoptotic signaling pathway via death domain receptors;GO:0097191 extrinsic apoptotic signaling pathway;GO:0007283 spermatogenesis
*DERL2*	Derlin 2	GO:1904153 negative regulation of retrograde protein transport, ER to cytosol;GO:1904293 negative regulation of ERAD pathway;GO:1904152 regulation of retrograde protein transport, ER to cytosol
*DICER1*	Dicer 1, ribonuclease III	GO:0032290 peripheral nervous system myelin formation;GO:0014040 positive regulation of Schwann cell differentiation;GO:0033168 conversion of ds siRNA to ss siRNA involved in RNA interference
*ERCC1*	ERCC excision repair 1, endonuclease non-catalytic subunit	GO:1905765 negative regulation of protection from non-homologous end joining at telomere;GO:1904431 positive regulation of t-circle formation;GO:1905764 regulation of protection from non-homologous end joining at telomere
*ETS1*	ETS proto-oncogene 1, transcription factor	GO:0045648 positive regulation of erythrocyte differentiation;GO:1904996 positive regulation of leukocyte adhesion to vascular endothelial cell;GO:0060055 angiogenesis involved in wound healing
*FBXO38*	F-box protein 38	GO:0002842 positive regulation of T cell mediated immune response to tumor cell;GO:0002840 regulation of T cell mediated immune response to tumor cell;GO:0002424 T cell mediated immune response to tumor cell
*GADD45GIP1*	GADD45G interacting protein 1	GO:0070126 mitochondrial translational termination;GO:0071850 mitotic cell cycle arrest;GO:0070125 mitochondrial translational elongation
*HIKESHI*	Heat shock protein nuclear import factor hikeshi	GO:1900034 regulation of cellular response to heat;GO:0006606 protein import into nucleus;GO:0034605 cellular response to heat
*IFT52*	Intraflagellar transport 52	GO:0035720 intraciliary anterograde transport;GO:0035735 intraciliary transport involved in cilium assembly;GO:0042733 embryonic digit morphogenesis
*LGALS8*	Galectin 8	GO:1904977 lymphatic endothelial cell migration;GO:0098792 xenophagy;GO:0036303 lymph vessel morphogenesis
*MRPS11*	Mitochondrial ribosomal protein S11	GO:0070126 mitochondrial translational termination;GO:0070125 mitochondrial translational elongation;GO:0042769 DNA damage response, detection of DNA damage
*MTF2*	Metal response element binding transcription factor 2	GO:0061086 negative regulation of histone H3-K27 methylation;GO:0061087 positive regulation of histone H3-K27 methylation;GO:0061085 regulation of histone H3-K27 methylation
*MYC*	MYC proto-oncogene, bHLH transcription factor	GO:0090096 positive regulation of metanephric cap mesenchymal cell proliferation;GO:0090095 regulation of metanephric cap mesenchymal cell proliferation;GO:0090094 metanephric cap mesenchymal cell proliferation involved in metanephros development
*NDUFA4*	NDUFA4 mitochondrial complex associated	GO:0006123 mitochondrial electron transport, cytochrome c to oxygen;GO:0006120 mitochondrial electron transport, NADH to ubiquinone;GO:0042775 mitochondrial ATP synthesis coupled electron transport
*NDUFB7*	NADH:ubiquinone oxidoreductase subunit B7	GO:0006120 mitochondrial electron transport, NADH to ubiquinone;GO:0042775 mitochondrial ATP synthesis coupled electron transport;GO:0042773 ATP synthesis coupled electron transport
*OGT*	O-linked N-acetylglucosamine (GlcNAc) transferase	GO:0061087 positive regulation of histone H3-K27 methylation;GO:0061085 regulation of histone H3-K27 methylation;GO:0043982 histone H4-K8 acetylation
*OSBPL8*	Oxysterol binding protein like 8	GO:0046326 positive regulation of glucose import;GO:0010891 negative regulation of sequestering of triglyceride;GO:0046628 positive regulation of insulin receptor signaling pathway
*PDCD10*	Programmed cell death 10	GO:1903588 negative regulation of blood vessel endothelial cell proliferation involved in sprouting angiogenesis;GO:0036481 intrinsic apoptotic signaling pathway in response to hydrogen peroxide;GO:0090051 negative regulation of cell migration involved in sprouting angiogenesis
*PRMT2*	Protein arginine methyltransferase 2	GO:0019919 peptidyl-arginine methylation, to asymmetrical-dimethyl arginine;GO:0035247 peptidyl-arginine omega-N-methylation;GO:0060765 regulation of androgen receptor signaling pathway
*PUS3*	Pseudouridine synthase 3	GO:0031119 tRNA pseudouridine synthesis;GO:1990481 mRNA pseudouridine synthesis;GO:0001522 pseudouridine synthesis
*RORA*	RAR related orphan receptor A	GO:0021702 cerebellar Purkinje cell differentiation;GO:0072539 T-helper 17 cell differentiation;GO:0021694 cerebellar Purkinje cell layer formation
*RPS15A*	Ribosomal protein S15a	GO:0006614 SRP-dependent cotranslational protein targeting to membrane;GO:0006613 cotranslational protein targeting to membrane;GO:0000184 nuclear-transcribed mRNA catabolic process, nonsense-mediated decay
*RPS29*	Ribosomal protein S29	GO:0006614 SRP-dependent cotranslational protein targeting to membrane;GO:0006613 cotranslational protein targeting to membrane;GO:0000184 nuclear-transcribed mRNA catabolic process, nonsense-mediated decay
*SLC35A2*	Solute carrier family 35 member A2	GO:0072334 UDP-galactose transmembrane transport;GO:0090481 pyrimidine nucleotide-sugar transmembrane transport;GO:0006012 galactose metabolic process
*SON*	SON DNA and RNA binding protein	GO:0048024 regulation of mRNA splicing, via spliceosome;GO:0000281 mitotic cytokinesis;GO:0050684 regulation of mRNA processing
*SPAG9*	Sperm associated antigen 9	GO:0007257 activation of JUN kinase activity;GO:0043507 positive regulation of JUN kinase activity;GO:0043506 regulation of JUN kinase activity
*TRRAP*	Transformation/transcription domain associated protein	GO:0043968 histone H2A acetylation;GO:0043967 histone H4 acetylation;GO:1904837 beta-catenin-TCF complex assembly
*TTC1*	Tetratricopeptide repeat domain 1	GO:0006457 protein folding;GO:0009987 cellular process;GO:0008150 biological_process
*TXNL1*	Thioredoxin like 1	GO:0045454 cell redox homeostasis;GO:0019725 cellular homeostasis;GO:0055114 oxidation-reduction process
*USP33*	Ubiquitin specific peptidase 33	GO:0071108 protein K48-linked deubiquitination;GO:0070536 protein K63-linked deubiquitination;GO:0051298 centrosome duplication
*ZDHHC3*	Zinc finger DHHC-type palmitoyltransferase 3	GO:1903546 protein localization to photoreceptor outer segment;GO:0097499 protein localization to non-motile cilium;GO:0018230 peptidyl-L-cysteine S-palmitoylation

### The 36 Shared DEGs Play an Essential Role in Endothelium Biology

**Figure 2:**
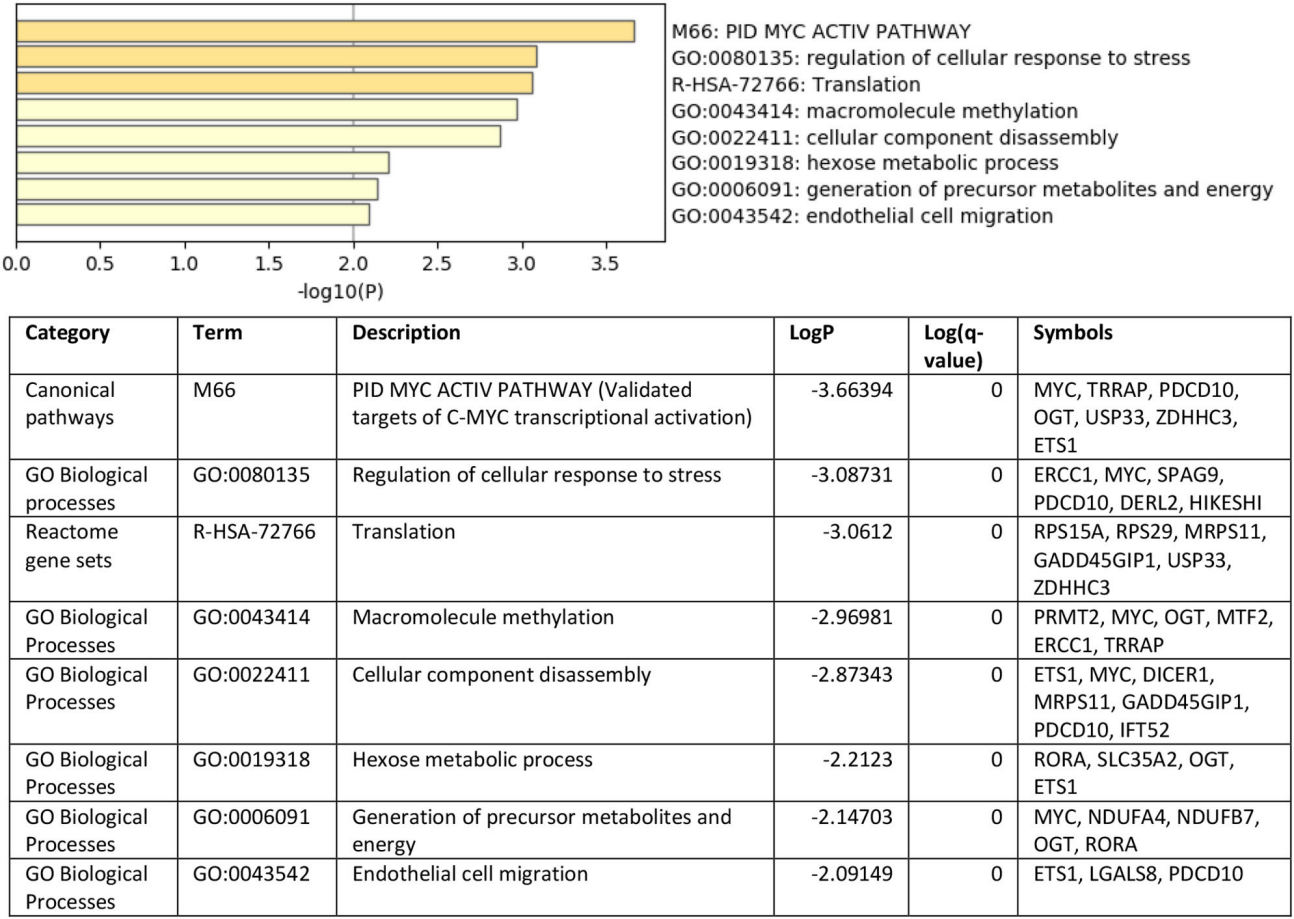
Top pathways enriched with shared DEGs in the blood and endothelium of patients with VTE compared to healthy controls. Created with BioRender.com.

To understand the role of the identified 36 genes, we explored their shared biological pathways and found that several of these genes were vital for pathways involved in cell homeostasis, response to stress, and cellular metabolism. These include pathways related to targets of C-MYC transcriptional activation (*MYC, TRRAP, PDCD10, OGT, USP33, ZDHHC3, and ETS1*), regulation of cellular response to stress (*ERCC1, MYC, SPAG9, PDCD10, DERL2*, and *HIKESHI*), and endothelial cell migration (*ETS1, LGALS8*, and *PDCD10*). Figure 2 shows the list of biological pathways associated with the DEGs. Four genes *MYC, ETS1, OGT*, and P*DCD10* were shown to be common between the top pathways indicating their significant molecular and biological role:. They are all enriched in the PID MYC ACTIV PATHWAY, suggesting that they are targets of C-MYC transcriptional activation. The proto-oncogene *c-Myc* is vital for vascular development. Gene expression analysis of *c-Myc*-deficient endothelial cells showed that the senescent phenotype of *c-Myc* is needed for the prevention of vascular pro-inflammatory phenotype (28). Global or endothelial and hematopoietic cell-specific loss of *c-Myc* leads to defects in vasculogenesis and primitive erythropoiesis (29).

### SON, OGT, and RORA Are Differentially Expressed in the Peripheral Blood of Patients With Acute Coronary Syndrome and Heart Failure

**Figure 3:**
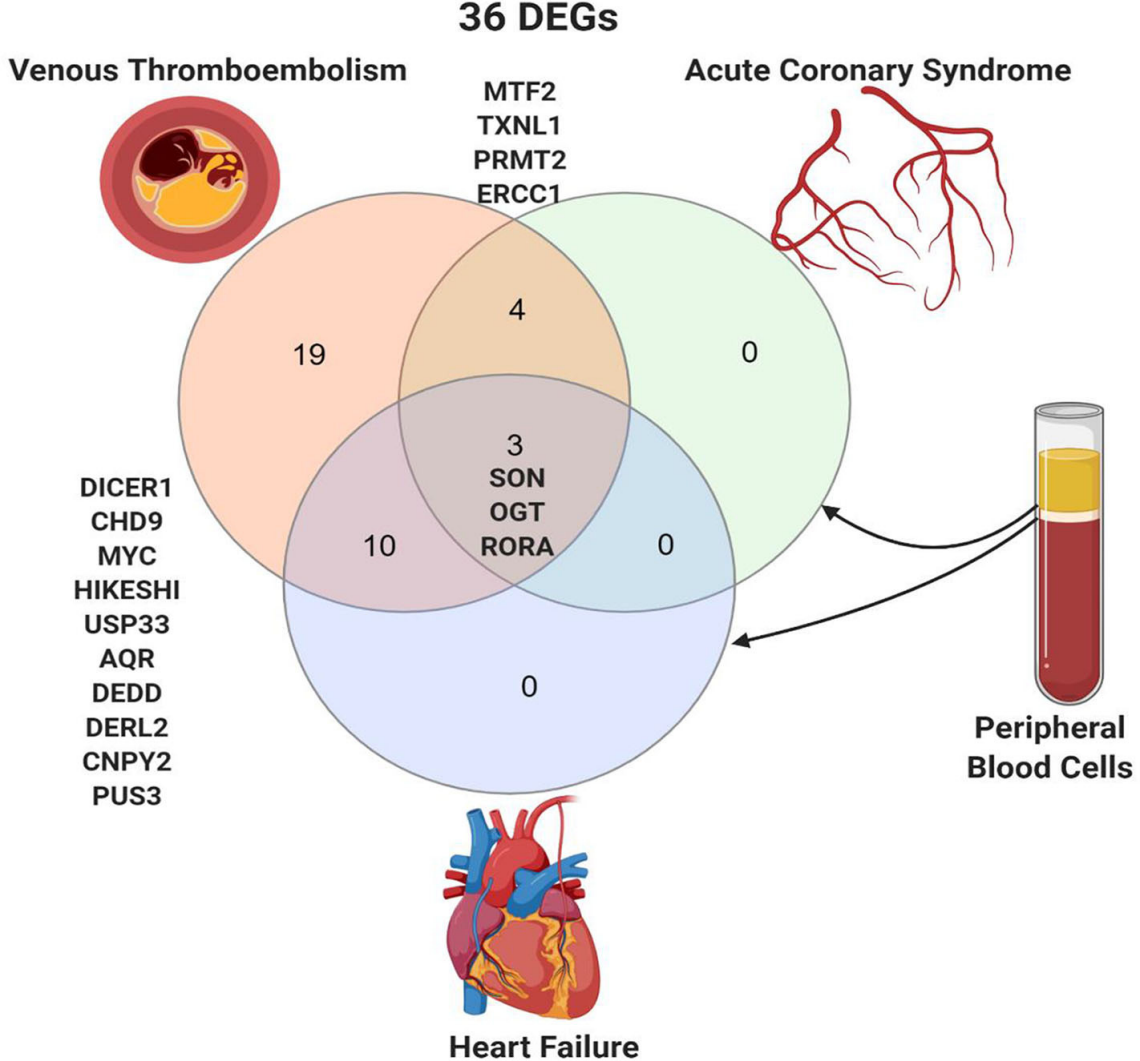
Shared peripheral blood DEGs in patients with venous thromboembolism, acute coronary syndrome, and heart failure. Created with BioRender.com.

The 36 genes identified to be specific to VTE were intersected with DEGs in thrombus-derived white blood cells of patients with acute coronary syndrome vs. controls (GSE19339) and peripheral blood mononuclear cells of patients with heart failure vs. control (GSE9128) (Figure 3). Four genes were shared between VTE and acute coronary syndrome (*MTF2, TXNL1, PRMT2*, and *ERCC2*), and ten genes were shared between VTE and heart failure (*DICER1, CHD9, MYC, HIKESHI, USP33, AQR, DEDD, DERL2, CNPY2*, and *PUS3*). Only three genes, *SON* (SON DNA and RNA binding protein), *OGT* (O-linked N-acetylglucosamine [GlcNAc] transferase), and *RORA* (RAR related orphan receptor A) were shared by all the three conditions.

### *SON, OGT*, and *RORA* Expression in Healthy Endothelium of African Americans

**Figure 4:**
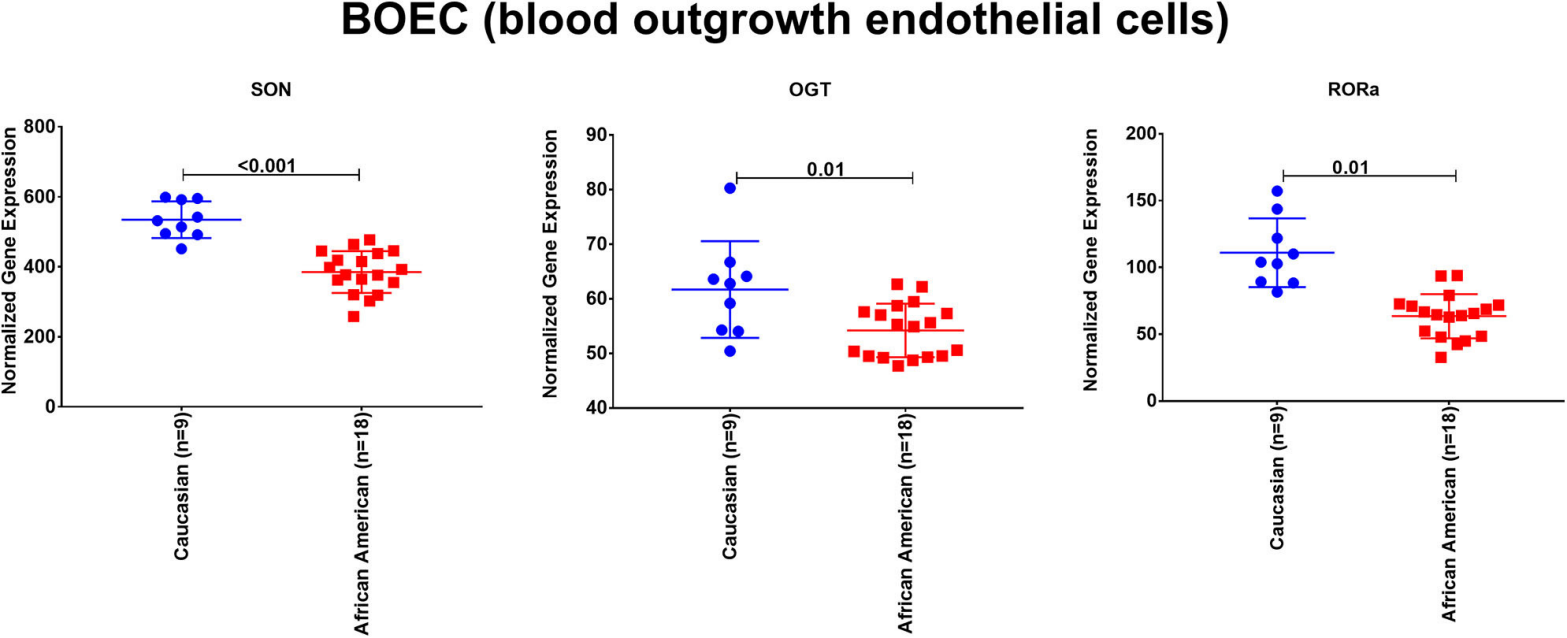
The mRNA expression of SON, OGT, and RORA in the publicly available dataset (GSE17078) of blood outgrowth endothelial cells from 27 healthy subjects of diverse ages and grouped into Caucasian and African Americans. Created with BioRender.com.

To explore the premise of genetic susceptibility for COVID-19 related cardiac events, we explored the gene expression of the three shared DEGs (*SON, OGT*, and *RORA*) in the publicly available dataset (GSE17078) of blood outgrowth endothelial cells from 27 healthy Caucasian and African American subjects. The findings show that *SON, OGT*, and *RORA* are significantly downregulated in the healthy endothelium of African Americans compared to Caucasians (Figure 4).

### Expression of the DEGs in Viral Infections

All 36 DEGs showed differential expression during viral infections as per the “Gene-virus associations by differential expression of gene following viral infection” database. The most frequently identified viruses affecting most of the genes were SARS-CoV strains from infected lung epithelial cells (SARS-CoV, SARS-dORF6, or SARS-BatSRBD “GSE47960, GSE47961 and GSE50000”; icSARS-CoV or the icSARS-dORF6 mutant “GSE37827”) and a SARS CoV MA15 infection in C57Bl/6 mouse model “GSE33266.” The genes which showed consistent differential expression in different datasets in response to SARS-CoV infections were *SON, OGT, PRMT2* (protein arginine methyltransferase 2), *TXNL1* (thioredoxin like 1), *CNPY2* (canopy FGF signaling regulator 2), *MRPS11* (mitochondrial ribosomal protein S11), *SPAG9* (sperm associated antigen 9), *MTF2* (metal response element-binding transcription factor 2), *CHD9* (chromodomain helicase DNA binding protein 9), and *RPS29* (ribosomal protein S29).

### Lung Single-Cell Expression of DEGs

**Figure 5:**
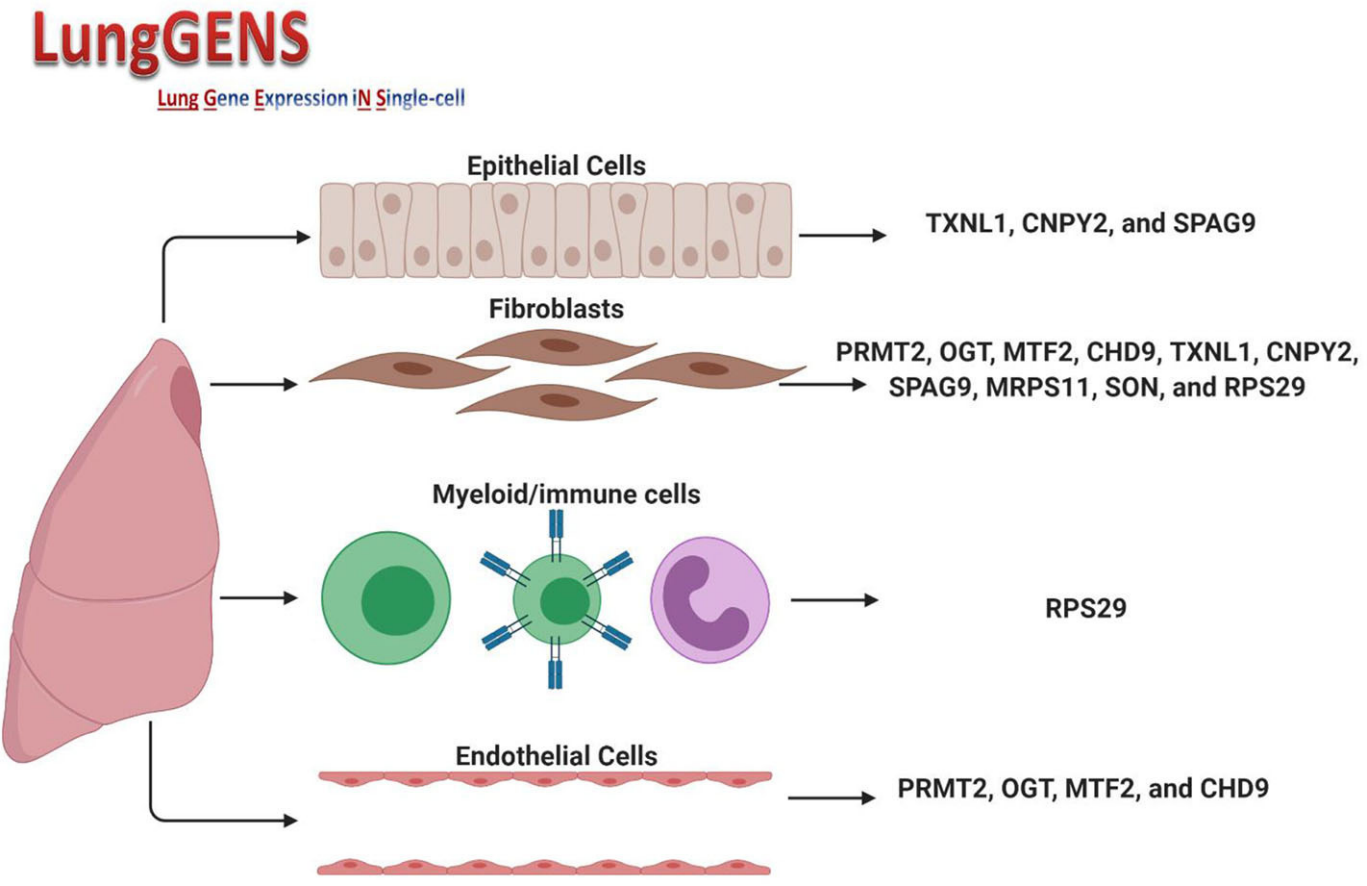
Expression of DEGs in different lung cell types. Created with BioRender.com.

The cellular composition of the lung is 40–50% endothelial cells, which differentiate in parallel with epithelial cells to form gas exchange units which are in contact with the external environment and thus need to ensure a rapid immune response (30). In lung diseases, including infections, the transcriptomes of endothelial cells, pericyte/smooth muscle cells, fibroblasts, and macrophage clusters showed that endothelial cells had the most differentially expressed gene profile compared to other cell types (31). We speculated that if we found common differentially expressed genes shared between the two cell types and which could be affected by SARS-CoV-2 infection, then we may be able further to understand the link between COVID-19 and associated endothelium injuries. Querying lung single-cell gene expression databases showed that expression of some of the 36 DEGs was significantly higher in lung endothelial cells (*PRMT2, OGT, MTF2*, and *CHD9*), lung fibroblasts (*PRMT2, OGT, MTF2, CHD9, TXNL1, CNPY2, SPAG9, MRPS11, SON*, and *RPS29*) and epithelial cells (*TXNL1, CNPY2*, and *SPAG9*). The only gene whose expression was found to also be related to myeloid/immune cells was *RPS29*. Figure 5 shows the DEGs and their expression in different cells. Details of peak expressions for each DEG in different cells is provided in Supplementary Table.

### SNPs in the Identified DEGs With Significant Association to COVID-19

We searched for the COVID-19 GWAS (https://grasp.nhlbi.nih.gov/Covid19GWASResults.aspx) looking for Annotated top results (only variants with P <1E-5) in 1,723 positive cases vs. 11,409 negative controls and found that none of the 36 genes identified carry SNPs with significant association to COVID-19, indicating that these genes are differentially expressed during infection or disease as a dynamic response to stimuli or condition.

### *RPS29* and *SPAG9* in SARS-CoV-2 Infected Lung Epithelial Cells

**Figure 6:**
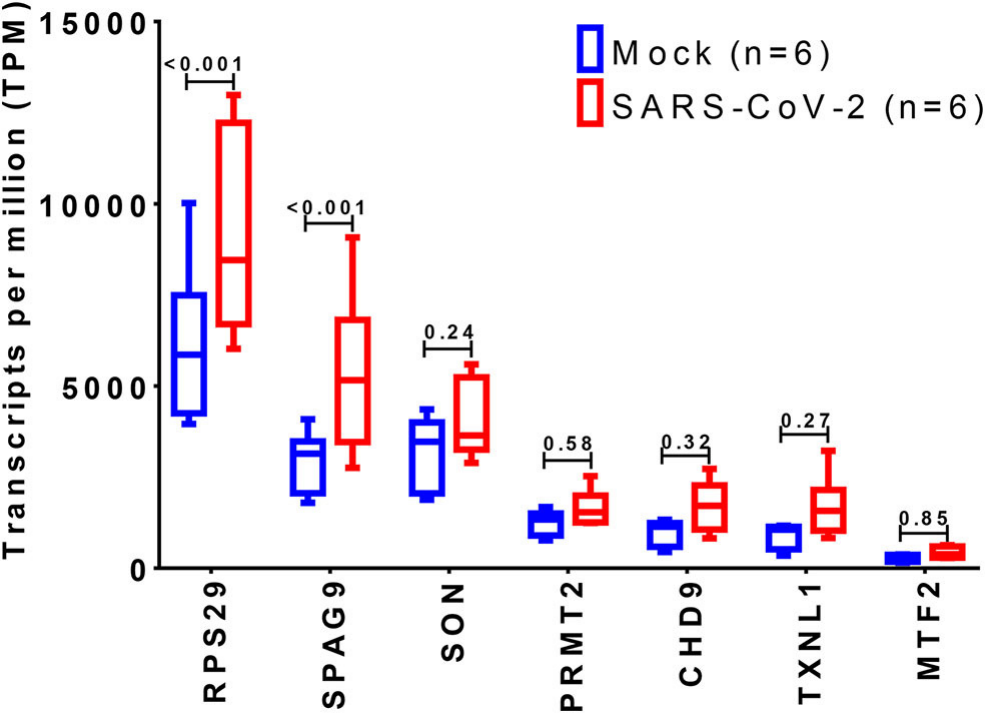
Identification of differentially expressed genes in SARS-CoV-2 infected cells. The shortlisted genes expression was explored in the dataset (GSE147507), where RNA-Sequencing of transformed alveolar lung cells (A549) were mock-treated (*n* = 6) or infected with SARS-CoV-2 (USA-WA1/2020) (*n* = 6). Created with BioRender.com.

The expression of the 36 DEGs was examined in mock vs. SARS-CoV-2 infected lung epithelial cells. Although most of the genes were upregulated by the virus infection, only *RPS29* and *SPAG9* showed significant upregulation, as shown in Figure 6.

### *SPAG9* and *RPS29* in Immune Cells

**Figure 7:**
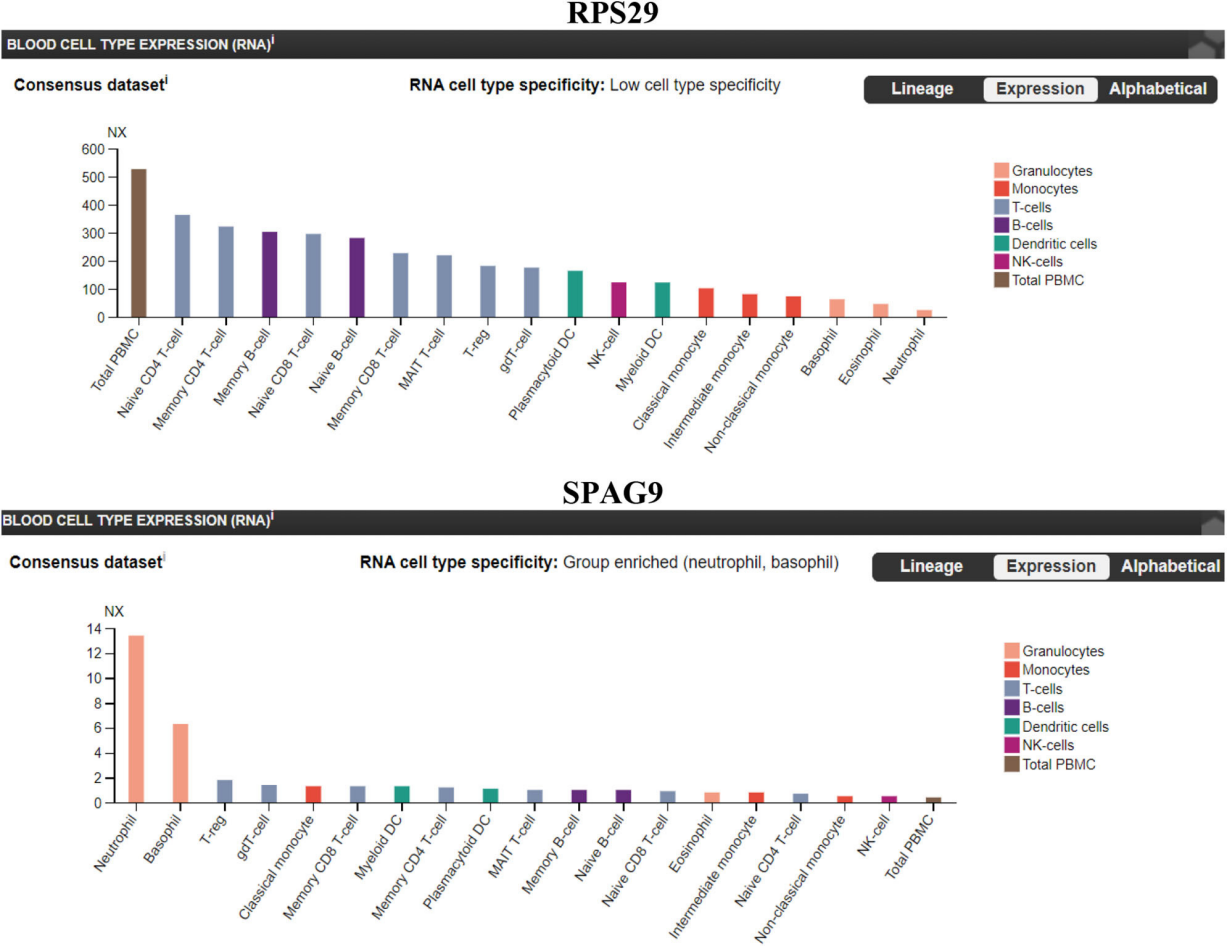
Immune cells specificity of the identified genes (RPS29 and SPAG9) using The Human Protein Atlas. A blood cell-type expression (RNA) option was used to examine the cell specificity of the identified genes. Normalized eXpression (NX) for 18 blood cell types and total peripheral blood mononuclear cells (PBMC) were explored. Created with BioRender.com.

We sought to identify which immune cell expresses the highest level of *RPS29* and *SPAG9*. Our findings indicate that *RPS29* showed low cell type specificity but was higher in T cells while *SPAG9* was enriched specifically in neutrophils and basophils (Figure 7).

### Cardiac Protective Genes Are Downregulated in Human-Induced Pluripotent Stem Cell-Derived Cardiomyocytes Infected With SARS-CoV-2

We explored the novel transcriptomic dataset “GSE150392” which is derived from *in vitro* work in which human-induced pluripotent stem cell-derived cardiomyocytes were infected with SARS-CoV-2. Nine of the 36 DEGs showed significant differential expression between SARS-CoV-2 and mock-infected cells (Table 3). Four genes (*NDUFA4, NDUFB7, MRPS11*, and *HIKESHI*) were downregulated by SARS-CoV-2 while the remaining five genes (*CHD9, MTF2, RORA, MYC*, and *ETS1*) were upregulated.

**Table 3:** List of genes significantly altered in human-induced pluripotent stem cell-derived cardiomyocytes infected with SARS-CoV-2 *in vitro* extracted from (GSE150392) dataset.

**Gene**	**logFC**	**AveExpr**	***t***	***P*-value**	**Adjusted *P*-value**	**B**
*NDUFA4*	−1.64768	8.542878	−3.32788	0.010298	0.05	−3.04532
*NDUFB7*	−1.60553	6.279966	−3.27608	0.011129	0.05	−3.1789
*MRPS11*	−1.44701	5.208024	−3.69074	0.006038	0.04	−2.54455
*HIKESHI*	−1.4191	5.802794	−3.37874	0.009546	0.05	−3.02343
*CHD9*	0.805961	6.451166	3.302155	0.010702	0.05	−3.13644
*MTF2*	0.810151	5.232699	3.511189	0.007846	0.04	−2.81722
*RORA*	1.288202	3.65585	4.223798	0.002848	0.02	−1.69915
*MYC*	1.848579	6.041511	6.609779	0.000162	0.01	1.25177
*ETS1*	1.886356	4.778408	4.258643	0.002716	0.02	−1.70091

## Discussion

Although respiratory failure has been the primary concern in COVID-19 infection, cardiac injury manifested by a rise in high-sensitivity troponin has gained considerable attention due to its reported association with mortality (3, 5). A higher incidence of acute onset heart failure, myocardial infarction, myocarditis, and cardiac arrest in COVID-19 patients is documented in the literature (9). On the basis of this, we hypothesized that a common molecular pathway shared between these common cardiovascular diseases might be activated in SARS-CoV-2 infection and thus provide an explanation for the high rate of cardiovascular complications seen in COVID-19 patients. To achieve that, we started by comparing cases to control in each of these diseases to find their shared DEGs, and then determine if these genes were also triggered specifically in COVID-19. The dataset we used for validation was Lung cells infected with SARS-CoV-2 (which is one of the few datasets available). As these identified genes were found to be expressed in lung cells, we postulate that they might represent the core machinery genes and the link between COVID-19, which is, in essence, lung infection and cardiovascular injuries, which are systemic consequences. While it would be ideal to utilize datasets derived from COVID-19 patients with cardiovascular outcomes for such comparative analysis, these are currently not available. Nevertheless, the findings from this study provide important new information that expands our current understanding of cardiovascular injuries in COVID-19.

From our comprehensive *in silico* approach, we identified 36 DEGs in the blood and endothelium of patients with VTE. Among these were genes known to play key roles in endothelium and vascular biology, with several being vital for pathways for C-MYC transcriptional activation, regulation of cellular response to stress as well as endothelial cell migration. In addition, some of the genes involved in endothelial cell migration (*ETS1, LGALS8*, and *PDCD10*) are also known to be associated with perturbations during viral infection (32–37). Notably, of the 36 DEGs identified, three genes, namely *SON, OGT*, and *RORA*, were also expressed in the peripheral blood of patients with acute coronary syndrome and heart failure. These findings implicate *SON, OGT*, and *RORA* as shared core genes in cardiac and vascular-related injuries. As these DEGs were also shared with mesenchymal cells of the lung, we speculate that they may represent the missing link between lung damage and related cardiovascular injuries reported in patients with COVID-19 patients. *SON* gene encodes an RNA-binding protein that promotes the splicing of many cell-cycle and DNA-repair transcripts and maintains accurate splicing for a subset of Human pre-mRNAs (38). *SON* is involved in pathways regulating virus infection like influenza virus infection as its deletion can lead to reduced influenza viral RNA levels and decreased viral infection suggesting that *SON* is needed for influenza virus replication (39). In human-induced pluripotent stem cell-derived multipotent cardiac progenitor cells, knockdown of *SON* reduced proliferation and differentiation of cardiomyocytes, while increasing fibroblasts (40). *OGT* is an O-GlcNAc transferase that catalyzes the addition of the O-GlcNAc post-translational modification to proteins, which is essential in regulating the stress response, differentiation, nutrient sensing, and autophagy (41). O-GlcNAc level is increased during ischemia-reperfusion or hemorrhagic shock with a cardioprotective effect making augmentation of O-GlcNAc levels a potential new therapeutic option for cardiovascular dysfunction or ischemia/reperfusion (42). *RORA* is a nuclear receptor retinoic acid-related orphan receptor-α that has been recently identified in the heart to inhibit ANG II-induced pathological hypertrophy and cardiomyocyte death, repress IL-6 transcription, and its level is reduced in failing mouse and human hearts (43). *RORA* deficient staggered mice subjected to myocardial ischemia/reperfusion injury show significantly increased myocardial infarct size, myocardial apoptosis, and exacerbated contractile dysfunction compared to wild-type mice (44). Moreover, mice with cardiomyocyte-specific *RORA* overexpression were less vulnerable to injury (44). *RORA* has been described as a transcription factor which ties metabolic and inflammatory signaling pathways. In fact, macrophages from staggerer mice (which have a deletion in *RORA*) overexpress *Il1b* following LPS stimulation suggesting an anti-inflammatory role for *RORA* (45). One mechanism that has been postulated involves the role of *RORA* in inducing IκBα, which negatively regulated the NFκB signaling pathway (46). However, it has been suggested that *RORA* may play a dual role in tissue and cell-dependent manner. For example, in adipose tissue *RORA* may play a pro-inflammatory role by driving endoplasmic reticulum stress (47). Interestingly in human-induced pluripotent stem, cell-derived cardiomyocytes infected *in vitro* with SARS-CoV-2, the expression of *RORA* was upregulated, and we speculate that this might be a cardioprotective response to direct viral invasion. Furthermore, *SON, OGT*, and *RORA* regulate the maintenance and differentiation of stem cells, including endothelial progenitor cells (EPCs) (6). They are preferentially expressed in undifferentiated stem cells but downregulated during stem cell differentiation (7–9). The ability of vascular endothelial cells to repair relies on the EPCs (11). As the occurrence of cardiovascular events during COVID-19 suggests that targeting the endothelium is part of the viral infection course, we surmise COVID-19 patients who have a pre-existing genetic propensity for low *SON, OGT*, and *RORA* expression may therefore be more susceptible to cardiac damage. Our findings of significant downregulation of *SON, OGT*, and *RORA* in healthy endothelium of African Americans is consistent with this hypothesis. This may explain the increased risk of cardiovascular injury among African American patients with COVID-19.

**Figure 8:**
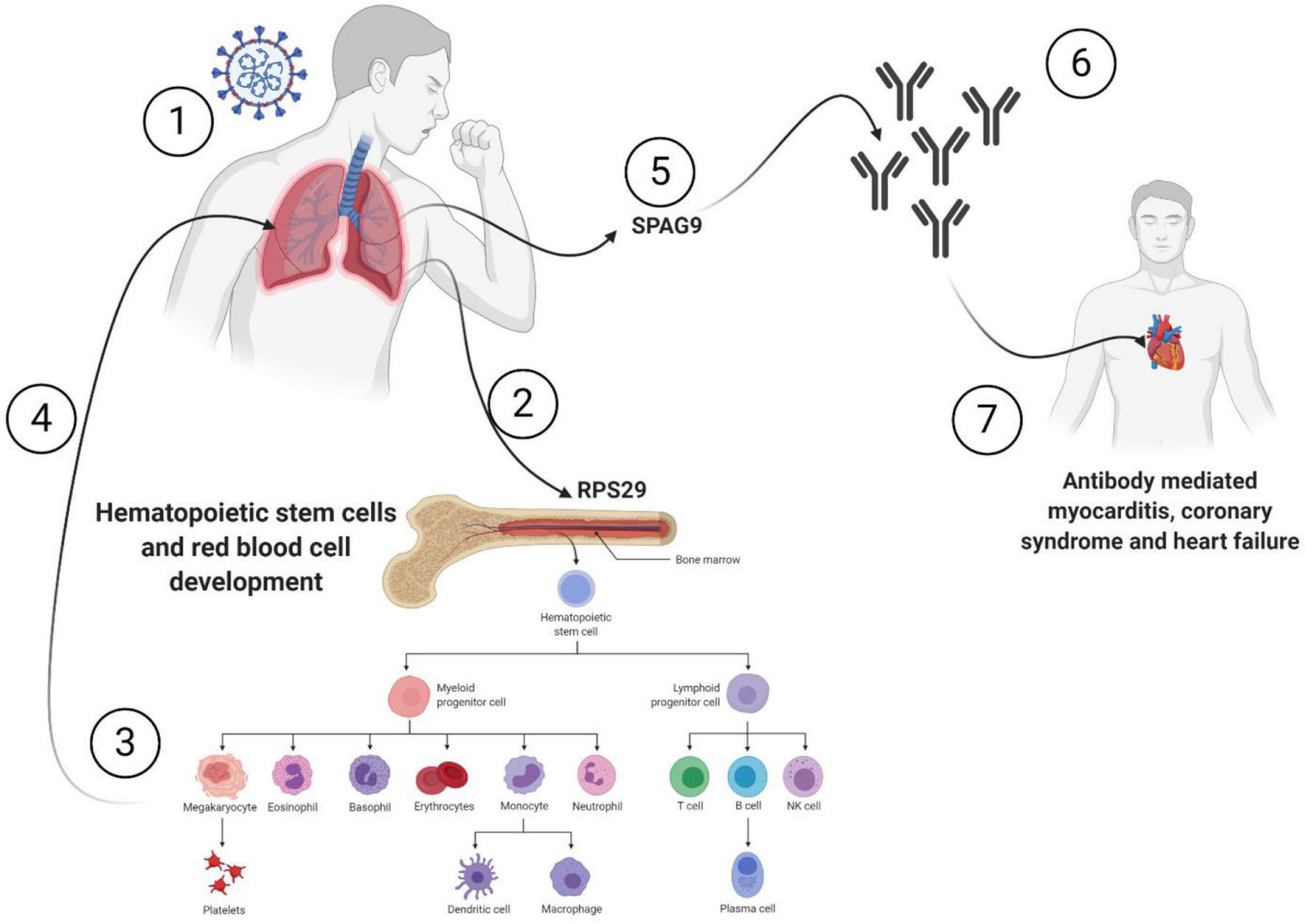
Role of *RPS29* and *SPAG9* genes in SARS-COV-2 related cardiovascular injuries. (1) Lung viral infection (2) upregulates *RPS29* that can (3) stimulate hematopoietic stem cells and red blood cell development to provide immune cells like neutrophils (4) to reach the lung, (5) the virus-induced upregulation of *SPAG9* might (6) induce antibodies against it that might cross-(7) react with the heart cytoskeleton and cause cardiac damage in the form of myocarditis and cardiac dysfunction. Created with BioRender.com.

All the 36 DEGs showed differential expression during viral infections, and the most frequently identified viruses were SARS-CoV strains. Specifically, in SARS-CoV-2 infected lung epithelial cells, *RPS29* and *SPAG9* genes were significantly upregulated. *RPS29*, which was the only DEG found to be specific to myeloid/immune Cells (S1.21) with TPM of 1,205.92 and intermediate fibroblast 2 (S2.5) with TPM of 1,098.99 in the lung, encodes for a ribosomal protein with an established role in hematopoietic stem cells and red blood cell development (48). *RPS29* is a component of the small 40S ribosomal subunit and needed for rRNA processing and ribosome biogenesis (49). Germ-line mutation in *RPS29* cause Diamond-Blackfan anemia, which is an inherited bone marrow failure syndrome (49). RNA-seq analysis of acute myocardial infarction samples has shown that RPS29 was one of the top upregulated genes (50). Interestingly, *RPS29* has been reported to be upregulated in A549 cells infected with the novel H3N2 Swine Influenza virus and the 2009 H1N1 pandemic Influenza virus (51). It was also upregulated in inflammatory conditions like periodontitis and associated with raised IFN-α (52). It is likely that the upregulation of *RPS20* in viral infection provides a mechanism for stimulation of hematopoietic stem cells and red blood cell development for increased production of immune cells like neutrophils for recruitment to the site of infection. *SPAG9* is known to induce an immune response and to regulate JNK and mitogen-activated protein kinases (MAPKs) signaling pathways, cell cycle progression, and matrix metalloproteinases (53). *SPAG9* is involved in the trafficking of endocytic vesicles within the intercellular bridge (54). *SPAG9* antibody in serum appears to be related to the type of lung cancer, indicating its specificity to lung-related tissues (55). It is one of the cardiac cytoskeleton and sarcomere assembly and function genes which are enhanced in mice with the deleted muscleblind-like family of splice regulators involved in cardiac dysfunction (56). The virus-induced upregulation of *SPAG9* might induce antibodies against it that might cross-react with the heart cytoskeleton and cause cardiac damage in the form of myocarditis and cardiac dysfunction. In Figure 8, we illustrate the pathway for the postulated role of *RPS29* and *SPAG9* genes in SARS-COV-2 related cardiovascular injuries.

Our analysis of the SARS-CoV-2 infected cardiomyocyte derived dataset showed that of the 36 DEGs identified in this study, four genes (*NDUFA4L2; NDUFB7; MRPS11; HIKESHI*) which are known to be cardioprotective were downregulated. *NDUFA4L2* plays a role in protecting cardiomyocytes from apoptosis and mitochondrial dysfunction during ischemia/reperfusion event, while *NDUFB7* has been linked with mitochondrial dysfunction and cardiomyocyte senescence (57, 58). *MRPS11* is a mitochondrial gene involved in sex-specific cardiac structure and function alterations (59). Heat shock proteins are involved in protecting the heart against heart failure by facilitating the removal of misfolded and degraded proteins (60), and *HIKESHI* plays a role in heat-shock stress response regulation to protect cells from heat shock damages. This finding suggests that in addition to the proposed *RPS29* and *SPAG9* induced cardiac damage pathway alluded to earlier, SARS-CoV-2 also employs a mechanism of downregulation of cardioprotective genes to promote cardiac injury.

In conclusion, our findings from the analysis of publicly available transcriptomic datasets identified three shared core genes pertinent to cardiac and vascular-related injuries. The possibility for their role in genetic susceptibility to cardiovascular injury in patients with COVID-19 was highlighted. In addition, it is likely that a combination of *RPS29* and *SPAG9* genes induced pathways, as well as downregulation of cardioprotective genes, contribute to cardiac and vascular events in patients with COVID-19.

Given that our analysis is *in silico*, experimental validation of our findings suggesting the potential role in genetic susceptibility such as *in vitro* experiments on endothelial cells exposed to SARS-CoV-2 antigens are needed to enable a better understanding of cardiovascular events associated with SARS-CoV-2 infection. The main limitation here is that the study is performed on the premise that venous thromboembolism, acute coronary syndrome, and heart failure might be common during COVID-19 infection. However, *in silico* analysis of studies with patients with COVID−19 infection vs. those without COVID-19 and a similar CVR outcome will be useful in pinpointing specific genes.

## Data Availability Statement

The raw data supporting the conclusions of this article will be made available by the authors, without undue reservation.

## Author Contributions

All authors listed have made a substantial, direct and intellectual contribution to the work, and approved it for publication.

## Conflict of Interest

The authors declare that the research was conducted in the absence of any commercial or financial relationships that could be construed as a potential conflict of interest.
