# Supplementary material for: Molecular Basis of Cardiac and Vascular Injuries Associated With COVID-19
Source: Front Cardiovasc Med. 2020 Nov 3;7:582399. doi: 10.3389/fcvm.2020.582399 (PMC7669624; doi:10.3389/fcvm.2020.582399)
Supplement: Supplementary file 1 [file Table_1.docx]

**Supplementary Table: Peaks of expression of DEGs in different lung cell types identified using LungGENS**

| **Gene Symbol** | **Description** | **LungGENS (Lung Gene Expression iN Single Cell** | |
| --- | --- | --- | --- |
|  |  | **Peak1 in:** | **Peak2 in:** |
| *PRMT2* | protein arginine methyltransferase 2 | **Endothelial Cells** (S2.46)  with TPM of 918.739 | **Intermediate Fibroblast 1** (S1.51)  with TPM of 246.833 |
| *OGT* | O-linked N-acetylglucosamine (GlcNAc) transferase | **Endothelial Cells** (S1.76)  with TPM of 401.748 | **Intermediate Fibroblast 2** (S1.57) with TPM of 318.759 |
| *MTF2* | metal response element binding transcription factor 2 | **Endothelial Cells** (S2.46)  with TPM of 191.776 | **Myofibroblast/SmoothMuscle** (S3.72)  with TPM of 150.976 |
| *CHD9* | chromodomain helicase DNA binding protein 9 | **Matrix Fibroblast** (S1.24)  with TPM of 67.5568 | **Endothelial Cells** (S1.25)  with TPM of 53.104 |
| *TXNL1* | thioredoxin like 1 | **Matrix Fibroblast** (S1.73)  with TPM of 182.21 | **Epithelial Cells** (S2.32)  with TPM of 137.111 |
| *CNPY2* | canopy FGF signaling regulator 2 | **Epithelial Cells** (S1.50)  with TPM of 392.634 | **Matrix Fibroblast** (S1.27)  with TPM of 322.491 |
| *SPAG9* | sperm associated antigen 9 | **Myofibroblast/SmoothMuscle** (S3.55)  with TPM of 64.7714 | **Epithelial Cells** (S2.26)  with TPM of 63.7232 |
| *MRPS11* | mitochondrial ribosomal protein S11 | **Intermediate Fibroblast 2** (S2.25)  with TPM of 280.24 | **Intermediate Fibroblast 2** (S1.85)  with TPM of 191.531 |
| *SON* | SON DNA and RNA binding protein | **Intermediate Fibroblast 2** (S1.57)  with TPM of 141.143 | **Intermediate Fibroblast 1** (S2.19)  with TPM of 125.345 |
| *RPS29* | ribosomal protein S29 | **Myeloid/Immune Cells**(S1.21) with TPM of 1205.92 | **Intermediate Fibroblast 2**(S2.5) with TPM of 1098.99 |
